# N^6^-methyladenosine (m^6^A) methyltransferase KIAA1429 accelerates the gefitinib resistance of non-small-cell lung cancer

**DOI:** 10.1038/s41420-021-00488-y

**Published:** 2021-05-17

**Authors:** Jun Tang, Tianci Han, Wei Tong, Jian Zhao, Wei Wang

**Affiliations:** 1 No. 1 Department of Tuberculosis, Chest Hospital, Shenyang, Liaoning Province 110044 China; 2 Department of Thoracic Surgery, Cancer Hospital of China Medical University, Shenyang, Liaoning Province 110042 China; 3 Department of Thoracic Surgery, Liaoning Cancer Hospital, Shenyang, Liaoning Province 110042 China

**Keywords:** Non-small-cell lung cancer, Cancer metabolism

## Abstract

N^6^-methyladenosine (m^6^A) modification has been convincingly identified to be a critical regulator in human cancer. However, the contribution of m^6^A to NSCLC gefitinib resistance is still largely unknown. Here, we screened and identified that m^6^A methyltransferase KIAA1429 was highly expressed in gefitinib-resistant NSCLC cells (PC9-GR), tissues, and closely related to unfavorable survival. Functionally, KIAA1429 accelerated the gefitinib resistance of NSCLC in vitro. Depletion of KIAA1429 repressed the tumor growth of PC9-GR cells in vivo. Mechanistically, KIAA1429 enhanced the mRNA stability of HOXA1 through targeting its 3′-untranslated regions (3′-UTR). Overall, our findings indicate that KIAA1429 plays essential oncogenic roles in NSCLC gefitinib resistance, which may provide a feasible therapeutic target for NSCLC.

## Introduction

Non-small-cell lung cancer (NSCLC) acts as the major subtype of lung cancer approximately 85%^1,2^. NSCLC is the most common malignancy and could give rise to tens of millions of death cases worldwide^3^. The main reason for this phenomenon is that chemotherapy drugs and targeted therapies lose their efficacy in clinical therapeutics^4^. Among these targeted therapy drugs, epidermal growth factor receptor tyrosine (EGFR) kinase inhibitors function as the first-line agent for advanced NSCLC patients harboring EGFR mutation, including gefitinib and erlotinib^5^. One of the drawbacks to note is that the chemotherapy resistance of gefitinib in NSCLC causes great therapeutic impedance^6^. Therefore, it is urgent to identify the oncogenic mechanism origin of gefitinib resistance in NSCLC.

N^6^-methyladenosine (m^6^A) is a reversible and dynamic process regulated by methyltransferases (writers), demethylases (erasers), and effector proteins (readers)^7–10^. Increasing evidence has indicated the roles of m^6^A key enzyme in human cancers. For example, m^6^A methyltransferase METTL14 is overexpressed in pancreatic cancer cells, which promotes the proliferation and migration via directly targeting PERP mRNA in an m^6^A-dependent manner^11^. Methyltransferase-like 3 (METTL3), METTL14, and Wilms tumor 1-associating protein (WTAP) construct a highly conserved multi-subunit methylase complex to mediate the conversion of adenosine to m^6^A. However, KIAA1429 is a subtype of m^6^A methyltransferase, which is well-known to all. In human cancer, KIAA1429 is found to be an oncogene. For example, KIAA1429 was upregulated in gastric cancer cells and tissues, which promotes the proliferation of gastric cancer cells. KIAA1429 regulates the c-Jun mRNA stability in an m^6^A-independent manner^12^. Therefore, these findings suggest that KIAA1429 may regulate the human tumorigenesis.

m^6^A methyltransferase KIAA1429, also known as VIRMA (vir-like m^6^A methyltransferase-associated protein), is a novel m^6^A methyltransferase complex component. In the present study, we aim to address the key regulation regarding the expression and mechanism of KIAA1429 in NSCLC gefitinib resistance. Incipiently, KIAA1429 was significantly upregulated in the NSCLC tissue samples. Thus, we carried out a series of assays to identify the functions of KIAA1429 in NSCLC tumorigenesis.

## Results

### m^6^A methyltransferase KIAA1429 was upregulated in lung cancer specimens

**Fig. 1:**
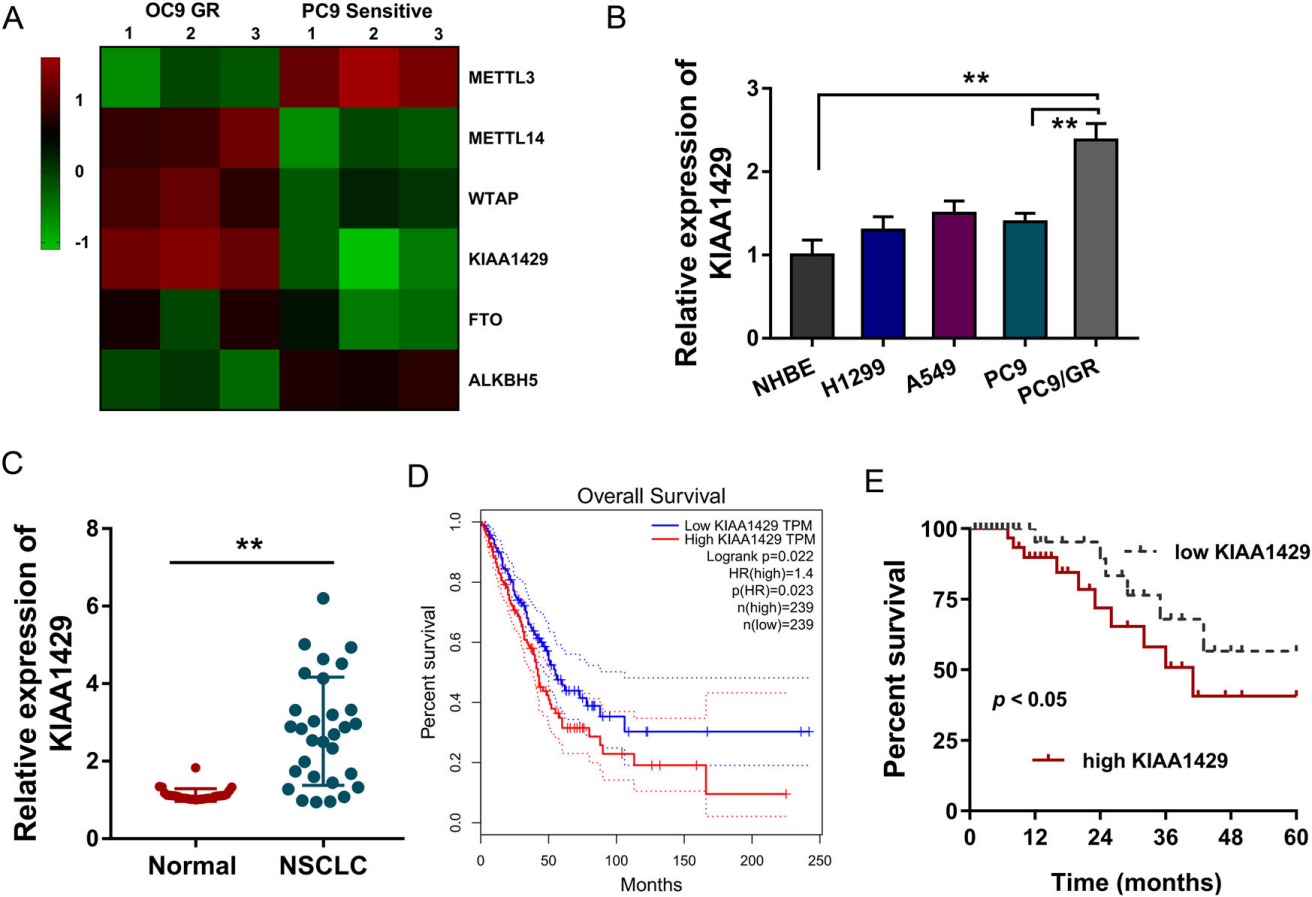
m^6^A methyltransferase KIAA1429 was upregulated in lung cancer specimens. **A** Heatmap showed the expression of several candidate m^6^A regulators in gefitinib-resistant NSCLC cell (PC9-GR) and parental sensitive cells (PC9). **B** RT-qPCR showed the KIAA1429 mRNA expression in the gefitinib-resistant NSCLC cell (PC9-GR) and other NSCLC cell lines (H1299, A549, and PC9). **C** RT-qPCR detected the KIAA1429 expression in NSCLC patients’ samples as compared to normal tissue samples. **D** Survival analysis based on TCGA database (http://gepia.cancer-pku.cn/) for lung cancer patients with high or low KIAA1429 expression. **E** Survival analysis based on clinical cohort for NSCLC patients with high or low KIAA1429 expression. Bar graphs indicate the means ± SD. *P < 0.05, **P < 0.01.

In the gefitinib-resistant NSCLC cell (PC9-GR), several candidate m^6^A regulators were detected as compared to the parental sensitive cells. Data illustrated that m^6^A methyltransferase KIAA1429 was remarkedly highly expressed in the PC9-GR cells (Fig. 1A). Moreover, in several NSCLC cell lines (H1299, A549, PC9, and PC9-GR), RT-qPCR showed that KIAA1429 mRNA was upregulated in the gefitinib-resistant NSCLC cell (PC9-GR) (Fig. 1B). In the NSCLC patients’ samples, KIAA1429 was significantly overexpressed as compared to normal tissue samples (Fig. 1C). The data on TCGA database (http://gepia.cancer-pku.cn/) showed that the patients with high KIAA1429 expression were tagged by lower survival percent (Fig. 1D). In addition to this, the survival analysis in the clinical cohort showed that the higher KIAA1429 expression was closely correlated with the lower survival of NSCLC patients (Fig. 1E). In conclusion, these findings implied that m^6^A methyltransferase KIAA1429 was upregulated in gefitinib-resistant NSCLC cells and closely correlated with the lower survival of NSCLC patients.

### KIAA1429 promoted the proliferation and gefitinib resistance of NSCLC cells

**Fig. 2:**
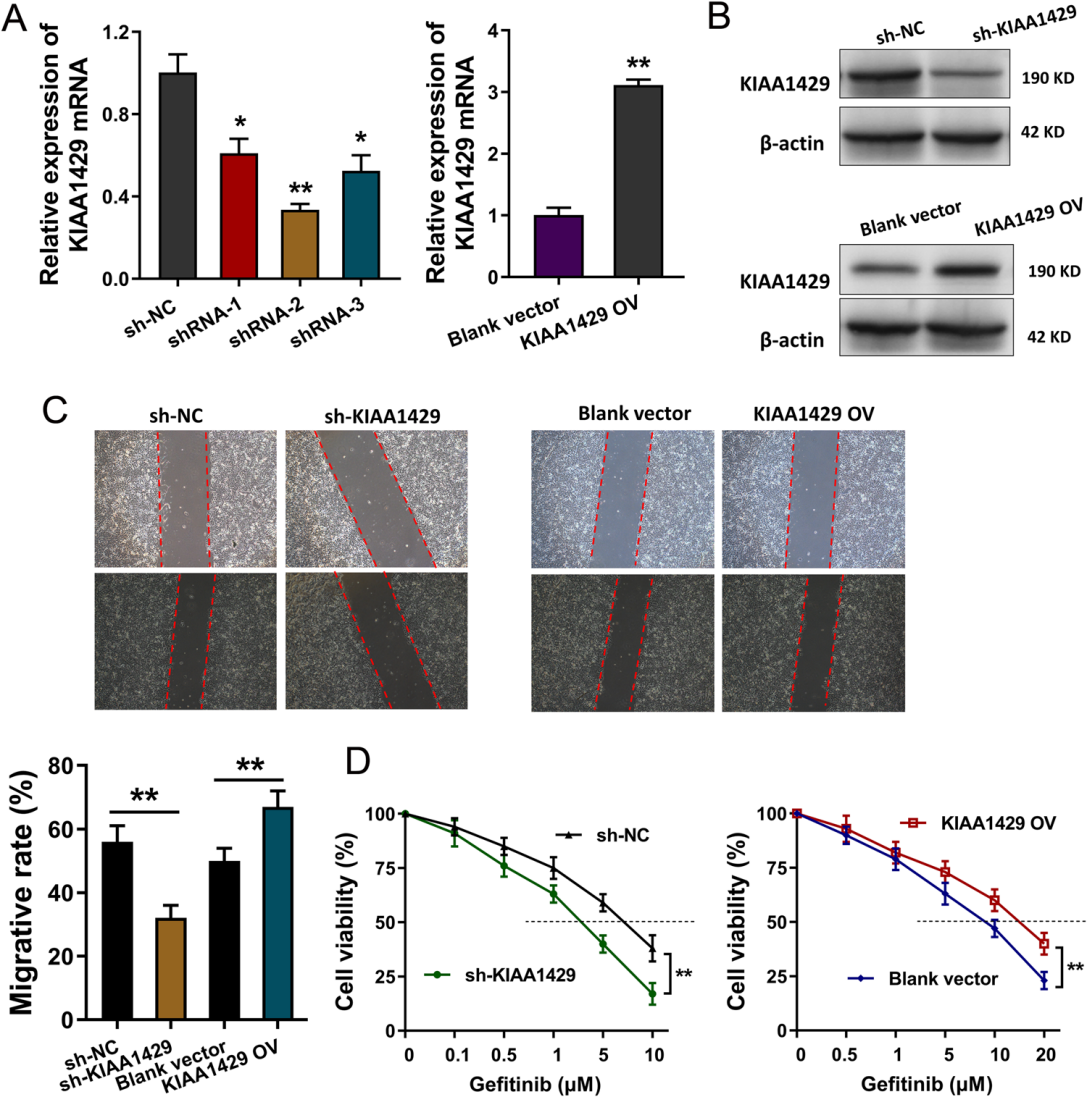
KIAA1429 promoted the proliferation and gefitinib resistance of NSCLC cells. **A** Short-hairpin RNA (shRNA) and overexpression plasmids were transfected in gefitinib-resistant NSCLC cells (PC9-GR) to construct the silencing and overexpression of KIAA1429. RT-qPCR was performed to detect KIAA1429 mRNA. **B** Western blot analysis detected the KIAA1429 protein level after silencing or overexpression plasmid transfection. **C** Wound-healing analysis detected the migrative ability of PC9-GR cells with KIAA1429 silencing or overexpression. **D** Gefitinib-resistant analysis was performed to detect the IC_50_ value (gefitinib concentration causing 50% absorbance decreasing). Bar graphs indicate the means ± SD. *P < 0.05, **P < 0.01.

In the gefitinib-resistant NSCLC cells (PC9-GR), the silencing and overexpression of KIAA1429 were constructed using short-hairpin RNA (shRNA) and overexpression plasmids. RT-qPCR demonstrated that KIAA1429 expression was remarkedly upregulated in PC9-GR cells (Fig. 2A). Western blot analysis found that KIAA1429 protein was significantly silenced or upregulated in the plasmid transfection (Fig. 2B). Wound-healing analysis found that KIAA1429 knockdown repressed the migrative ability and KIAA1429 overexpression accelerated the migrative ability (Fig. 2C). Gefitinib resistant analysis demonstrated that KIAA1429 knockdown reduced the IC_50_ value (inhibitory concentration 50) of PC9-GR cells, and KIAA1429 overexpression enhanced the IC_50_ value of PC9-GR cells (Fig. 2D). In conclusion, our findings suggested that KIAA1429 promoted the proliferation and gefitinib-resistant NSCLC cells.

### MeRIP-Seq revealed the m^6^A profile in gefitinib-resistant NSCLC cells

**Fig. 3:**
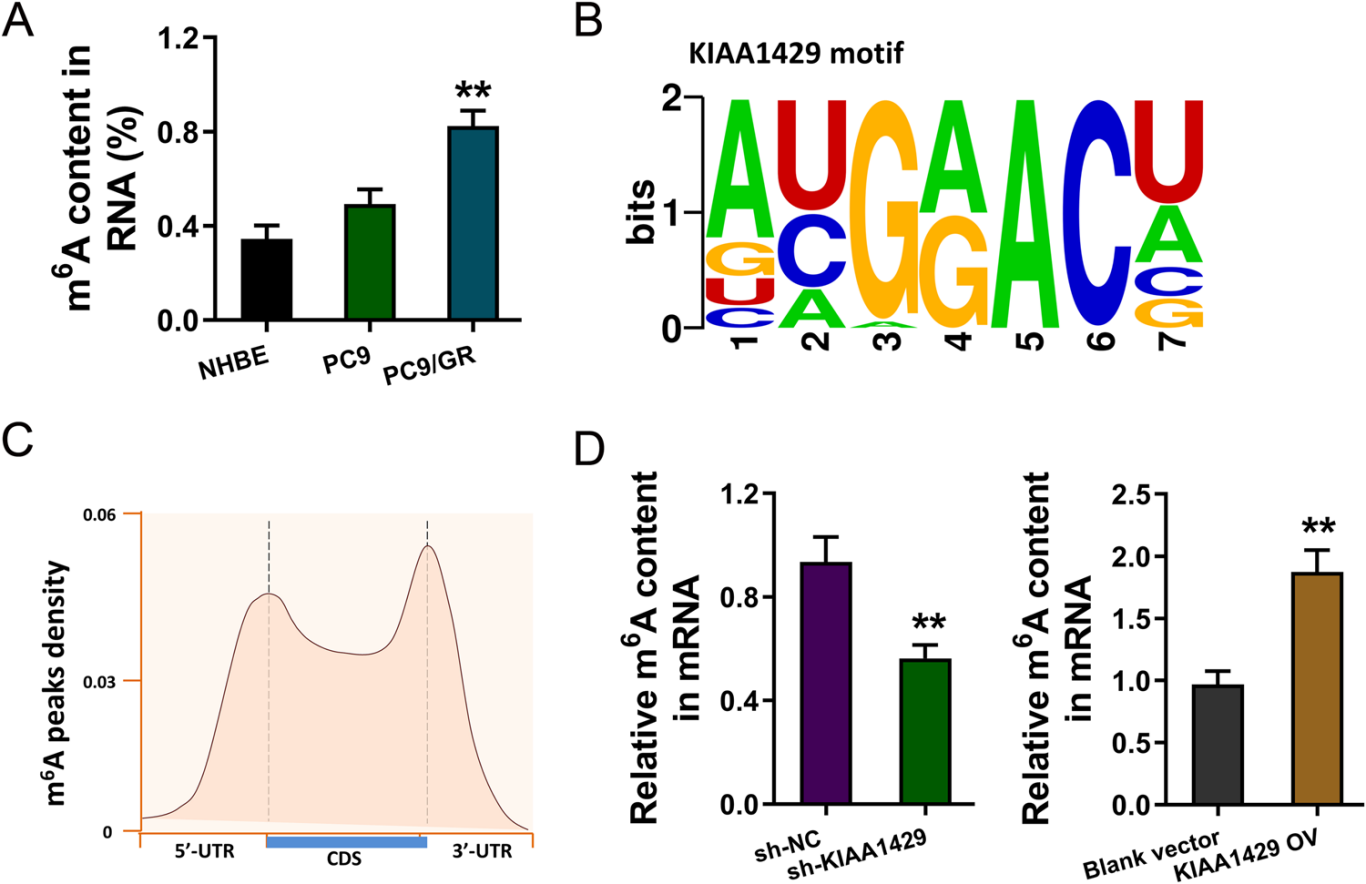
MeRIP-Seq revealed the m^6^A profile in gefitinib-resistant NSCLC cells. **A** m^6^A quantitative analysis was carried out to illustrate m^6^A enrichment in the gefitinib-resistant cells (PC9-GR) and parental cells (PC9). **B** The motif of KIAA1429 was identified (AUGGACU) according to MeRIP-Seq. **C** Metagene profile of m^6^A distribution across transcriptome was detected by this MeRIP-Seq. **D** m^6^A quantitative analysis was measured in PC9-GR cells with KIAA1429 knockdown and KIAA1429 overexpression transfection. Bar graphs indicate the means ± SD. **P < 0.01.

Recent research illustrated the increasing role of m^6^A in human cancer; thus, the present study focused on the potential regulation of KIAA1429 on NSCLC gefitinib resistance. m^6^A quantitative analysis was carried out, showing higher m^6^A enrichment in the gefitinib-resistant cells (PC9-GR) (Fig. 3A). To discover the m^6^A profile in the gefitinib-resistant NSCLC cells, MeRIP-Seq was performed in PC9-GR and PC9 cells. The motif of KIAA1429 was identified (AUGGACU) (Fig. 3B). Metagene profile of m^6^A distribution across the transcriptome was detected by this MeRIP-Seq (Fig. 3C). Moreover, KIAA1429 knockdown repressed the m^6^A level in PC9-GR cells, while KIAA1429 overexpression upregulated the m^6^A level (Fig. 3D). In summary, these findings suggested that MeRIP-Seq revealed the m6A profile in gefitinib-resistant NSCLC cells.

### KIAA1429 enhanced the stability of HOXA1 mRNA

**Fig. 4:**
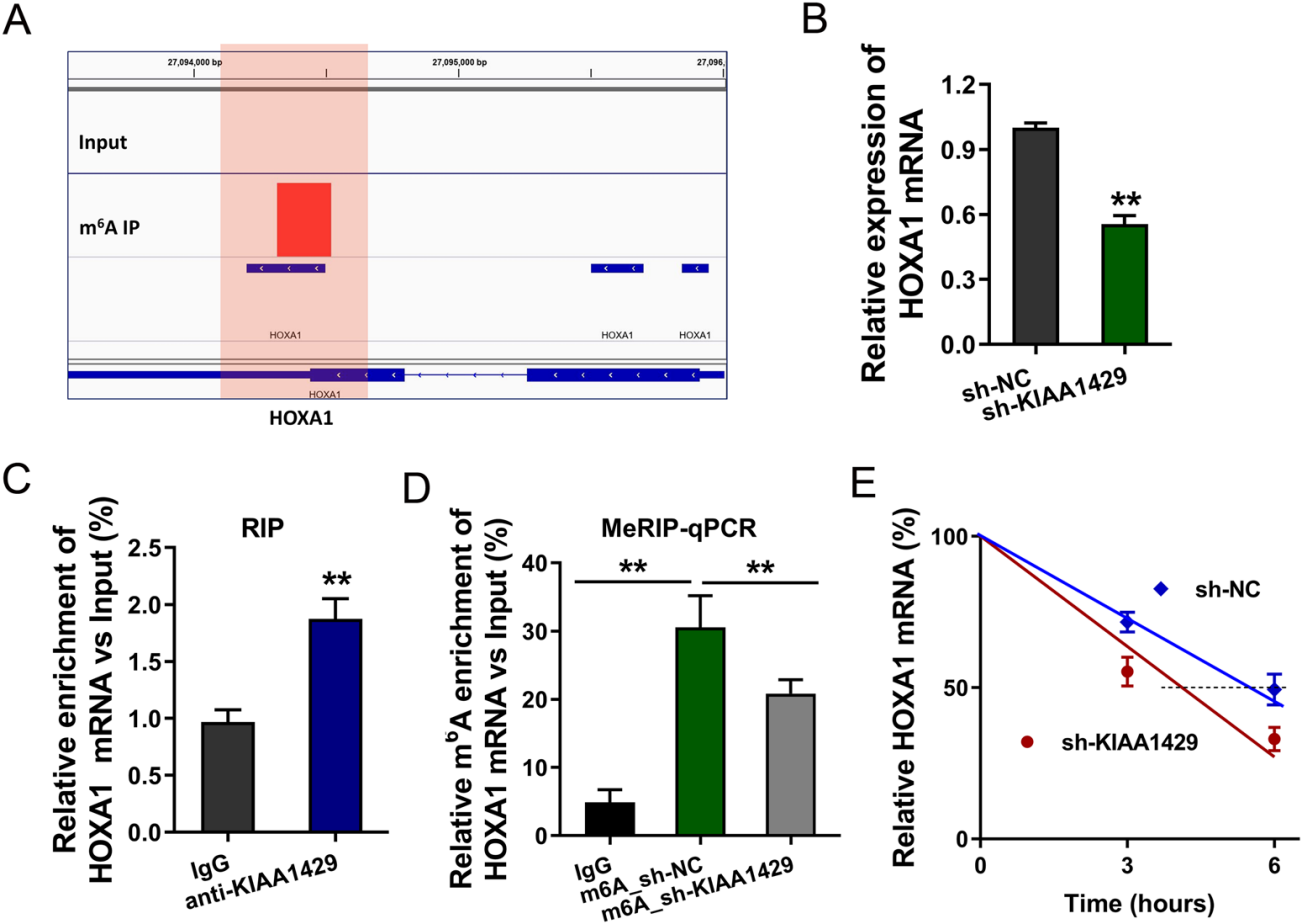
KIAA1429 enhanced the stability of HOXA1 mRNA. **A** Integrative genomics viewer (IGV) tool showed the remarkable m^6^A peak in the 3′-UTR of HOXA1 mRNA. **B** RT-qPCR was performed to detect the HOXA1 mRNA expression. **C** RNA-binding protein immunoprecipitation assay (RIP) demonstrated the interaction within KIAA1429 antibody (anti-KIAA1429) and m^6^A-modified HOXA1 mRNA. **D** MeRIP-qPCR with specific primers (HOXA1 mRNA primer) revealed the m^6^A modification level upon KIAA1429 knockdown or control. **E** KIAA1429-knockdown PC9-GR cells or control cells were treated with actinomycin D (1 μg/ml). The RNA decay rate was identified using qPCR normalized to initial 0-h expression. Bar graphs indicate the means ± SD. **P < 0.01.

Based on the MeRIP-Seq, we found that HOXA1 functioned as one of the targets of KIAA1429. Integrative genomics viewer (IGV) tool showed that there was a remarkable m^6^A peak in the 3′-UTR of HOXA1 mRNA (Fig. 4A). In the PC9-GR cells, the HOXA1 mRNA expression was detected using qPCR, suggesting that HOXA1 mRNA level was statistically decreased upon KIAA1429 knockdown (Fig. 4B). Then, we performed RNA-binding protein immunoprecipitation assay (RIP) and data demonstrated that the KIAA1429 antibody could effectively combine with the HOXA1 mRNA, illustrating the interaction within KIAA1429 and m^6^A-modified HOXA1 mRNA (Fig. 4C). To further demonstrate the role of KIAA1429 on HOXA1 m^6^A modification, MeRIP-qPCR with specific primers revealed that m^6^A modification level was reduced upon KIAA1429 knockdown (Fig. 4D). Interestingly, we also observed the HOXA1 mRNA stability decreasing upon KIAA1429 knockdown (Fig. 4E). Taken together, HOXA1 acted as a downstream target of KIAA1429, and KIAA1429 enhanced the stability of HOXA1 mRNA.

### KIAA1429/HOXA1 axis promoted the proliferation and gefitinib resistance of NSCLC cells

**Fig. 5:**
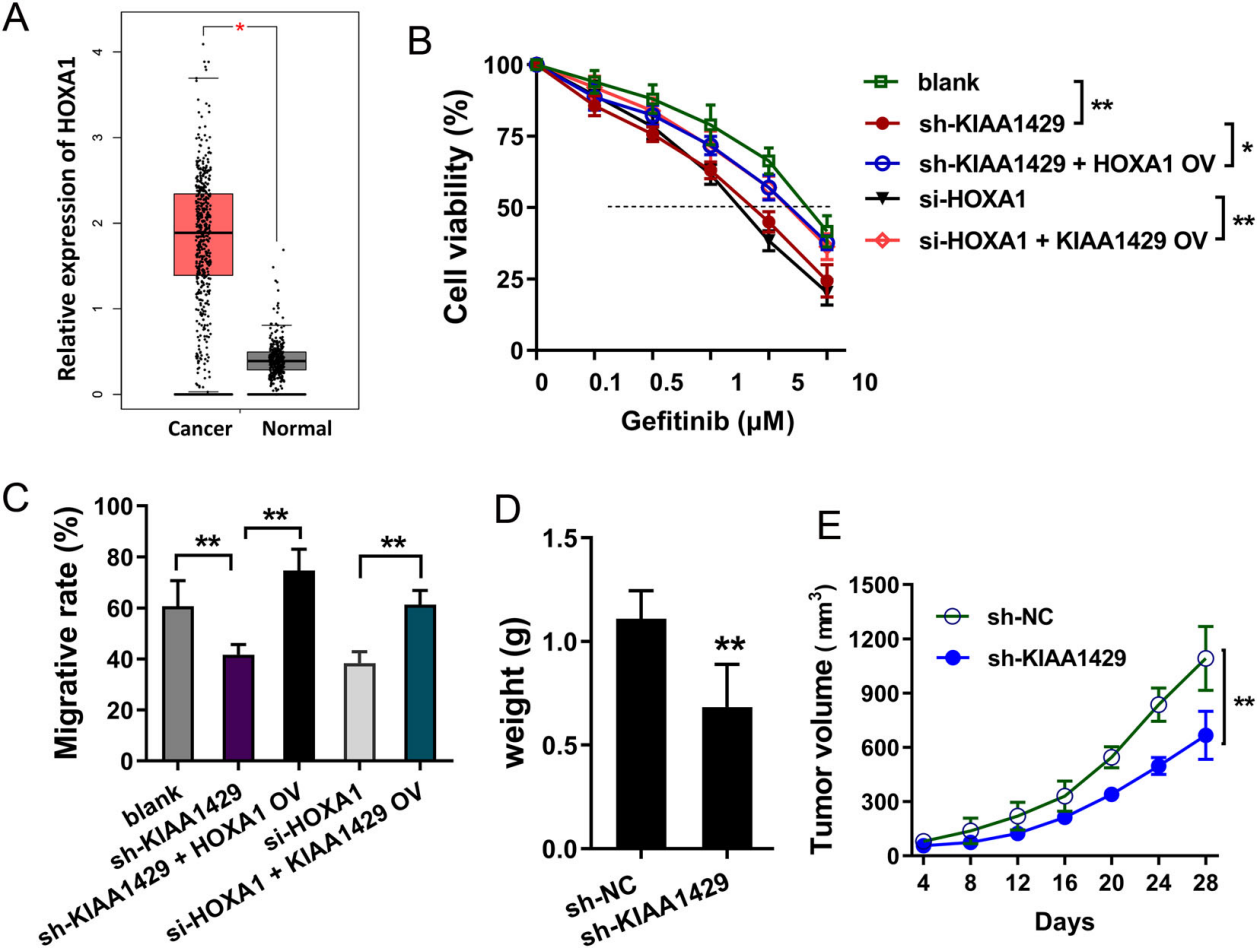
KIAA1429/HOXA1 axis promoted the proliferation and gefitinib resistance of NSCLC cells. **A** HOXA1 expression was upregulated in the lung cancer cohort based on the TCGA database (http://gepia.cancer-pku.cn). **B** Gefitinib-resistant analysis was performed to detect the IC_50_ value (gefitinib concentration causing 50% absorbance decreasing) in PC9-GR cells that transfected with KIAA1429 knockdown (sh-KIAA1429), HOXA1 overexpression (HOXA1 OV), HOXA1 knockdown (si-HOXA1), and KIAA1429 overexpression plasmid (KIAA1429 OV). **C** Wound-healing analysis was conducted using PC9-GR cells transfected with KIAA1429 knockdown (sh-KIAA1429), HOXA1 overexpression (HOXA1 OV), HOXA1 knockdown (si-HOXA1), and KIAA1429 overexpression plasmid (KIAA1429 OV). **D** Tumor weight and (**E**) volume were detected using PC9-GR cells transfected with KIAA1429 knockdown (sh-KIAA1429) and control (sh-NC). Bar graphs indicate the means ± SD. *P < 0.05, **P < 0.01.

Based on the TCGA database (http://gepia.cancer-pku.cn), HOXA1 expression was found to be upregulated in the lung cancer cohort (Fig. 5A). Gefitinib-resistant analysis demonstrated that KIAA1429 knockdown (sh-KIAA1429) or HOXA1 knockdown (si-HOXA1) reduced the IC_50_ value (inhibitory concentration 50) of PC9-GR cells, and KIAA1429 overexpression or HOXA1 overexpression (HOXA1 OV) rescued the IC_50_ value of PC9-GR cells (Fig. 5B). Wound-healing analysis found that KIAA1429 or HOXA1 knockdown repressed the migrative ability, while HOXA1 or KIAA1429 overexpression recovered the migrative ability repressed by HOXA1 knockdown (Fig. 5C). In vivo xenograft assay showed that the tumor weight and volume were both inhibited upon KIAA1429 knockdown in PC9-GR cells (Fig. 5D, E). In summary, our findings suggested that KIAA1429/HOXA1 axis promoted the proliferation and gefitinib resistance of NSCLC cells.

## Discussion

Emerging research has indicated that the dysregulation of epigenetic modifications has played an increasingly important role in human cancers^13,14^. RNA modification, especially m^6^A modification, is identified to participate in the NSCLC chemotherapy resistance^15,16^. Present research focuses on the potential regulation of m^6^A methyltransferase KIAA1429 toward the gefitinib resistance of NSCLC.

In spite of the fact that the oncogenic or antigenic modulations of m^6^A key regulators for human cancer have been elaborated, the deepgoing functions on another important layer are still bewildering^17–19^. Apart from DNA modification, the m^6^A modification that occurred on RNA has been recently proposed regarding epigenetic regulation, including cell differentiation, RNA splicing, and protein translation. In lung cancer, METTL3 expression, as well as m^6^A RNA modification, was remarkedly increased in TGF-β-induced epithelial−mesenchymal transition of lung cancer cells. METTL3 positively regulates the mRNA stability of JUNB mRNA and m^6^A modification enrichment^20^. Moreover, m^6^A demethylase ALKBH5 expression was elevated in lung adenocarcinoma, and the m^6^A level was upregulated in forkhead box M1 (FOXM1) mRNA. ALKBH5 downregulates the m^6^A modification enrichment of FOXM1 mRNA, thereby increasing FOXM1 expression^21^. Therefore, these findings identify that m^6^A regulators could remarkably regulate the tumorigenesis.

Here, we found that m^6^A methyltransferase KIAA1429 was upregulated in the gefitinib-resistant NSCLC cells (PC9-GR). Clinically, elevated KIAA1429 expression indicated the unfavorable outcome of NSCLC patients. Thus, this high-expressed KIAA1429 might function as an oncogenic indicator for NSCLC sufferers. In cellular experiments, gain-of-experiments and loss-of-functions were conducted and found that KIAA1429 promoted the migration of PC9-GR. Besides, gefitinib-sensitive assays found that KIAA1429 elevates the IC_50_ value (inhibitory concentration 50) of PC9-GR cells. These findings forcefully prove that KIAA1429 may regulate the gefitinib chemotherapy resistance potential in NSCLC cells.

Apart from the acceleration that KIAA1429 elevates the gefitinib resistance found in present research, more similar findings also evidence the potential function of m^6^A in human tumor chemotherapy-resistant. In hepatocellular carcinoma (HCC), m^6^A methyltransferase is significantly downregulated in sorafenib-resistant HCC cells. METTL3 knockdown promotes sorafenib resistance and angiogenesis gene expression and activates autophagy-associated pathways. METL3 enhances the 3′-untranslated region (3′-UTR) m^6^A modification of FOXO3 mRNA to increase its mRNA stability through YTHDF1-dependent manner^22^. In testicular germ cell tumors, METTL3 promotes the m^6^A-modified transcription factor-activating enhancer-binding protein 2C (TFAP2C) mRNA to regulate cisplatin treatment^23^. Overall, these data suggest that m^6^A modification could remarkably regulate the tumor chemotherapy resistance.

In present research, KIAA1429, which acts as a m^6^A methyltransferase, could target the 3′-UTR of HOXA1 mRNA. KIAA1429 could regulate the pathogenesis in human pathophysiological process. In oocyte, its growth is accompanied by the accumulation of post-transcriptional regulation and abundant RNAs’ modification. Loss of KIAA1429 leads to abnormal RNA metabolism in oocytes and modulates follicular development^24^. In gastric cancer, KIAA1429 is upregulated in tissue and cells, and the upregulated KIAA1429 promotes the proliferation by stabilizing c-Jun mRNA via m^6^A-independent manner^25^. Therefore, the regulation of KIAA1429 on NSCLC gefitinib resistance makes a great sense.

In summary, the present research found that KIAA1429 was upregulated in the gefitinib-resistant NSCLC cells and indicates the unfavorable outcome. KIAA1429 promotes the gefitinib resistance of NSCLC cells and mechanistically enhances the mRNA stability of HOXA1. Overall, these findings convincingly provide a druggable target for NSCLC patients.

## Materials and methods

### Clinical specimens

In total, 30 patients, who underwent surgical resection and were diagnosed as NSCLC, were recruited in the retrospective study between years 2017 and 2018. This research was approved by the Ethics Committee of Cancer Hospital of China Medical University. Written informed consents were obtained from all patients. Tumor tissue and the corresponding fresh tumor tissue samples were obtained from the tissue. Data regarding SCLC clinicopathological features were recorded shown in Table 1.

**Table 1:** Clinicopathological feature of NSCLC patients with KIAA1429 expression.

		KIAA1429		*p*
		Low (15)	High (15)	
Gender				
Male	16	7	9	0.498
Female	14	8	6	
Age (Year)				
≥60	15	6	9	0.736
<60	15	9	6	
TNM				
I−II	10	6	4	0.007*
III−IV	20	9	11	
Differentiation				
Well/moderate	14	7	7	0.326
Poor	16	8	8	
Lymph metastasis				
No	13	5	8	0.176
Yes	17	10	7	

^*^P < 0.05: statistical difference.

### Cell lines and culture condition

Human normal bronchial epithelial cells (NHBE) and NSCLC cell lines (PC9, gefitinib-resistant PC9/GR) were purchased from Cell Bank of Shanghai Institute of Biochemistry and Cell Biology (Shanghai, China). Cell lines were cultured in DMEM (Gibco, Waltham, MA, USA) supplemented with 10% FBS (Gibco), 1% mixture of penicillin, 25 mmol/L glucose, and streptomycin in a 5% CO_2_ incubator at 37 °C.

### Cell transfection

To overexpress KIAA1429, the full length of KIAA1429 cDNA (gene ID: NM_015496) was amplified and cloned into the lentivirus vector pLenti-copGFP-P2A-Puro-CMV-MCS-3Flag (GeneCopoeia, Madison, WI, USA). Lentivirus vectors containing KIAA1429 shRNA were produced (GenePharma Tech, Shanghai, China). For the transient transfection, siRNA targeting HOXA1 was designed and produced by RiboBio (Guangzhou, China) and transfected with Lipofectamine 2000 reagent (Invitrogen, Carlsbad, CA, USA), according to the manufacturer’s instructions.

### Real-time PCR

Total RNA was isolated from NSCLC tissue and cells using a miRNeasy Mini Kit (Qiagen, Hilden, Germany). The concentration and purity of the extracted RNA were determined using ultraviolet spectrophotometry (Nanodrop ND2000, Thermo Fisher Scientific). In all, 1 μg of total RNA was reverse-transcribed to cDNA using High Capacity RNA cDNA Kit (Applied Biosystems, Carlsbad, CA, USA). The mRNA levels of m^6^A writers (KIAA1429) and target protein (HOXA1) were determined using SYBR Green PCR Master Mix (Applied Biosystems) on an ABI7500 Real-time for real-time PCR analysis with primers (Supplementary Table S1). *β*-actin acted as the internal control in RT-qPCR assays. Real-time PCR was calculated using 2^−ΔΔCt^ method and normalized for relative gene expression.

### Western blotting

Protein samples were extracted from NSCLC cells using radio-immunoprecipitation assay (RIPA) lysis buffer (Sigma-Aldrich, St Louis, MO, USA) containing protease inhibitor cocktail. The protein concentration was determined by bicinchoninic acid (BCA) kit with deionized water. The SDS-PAGE (10%, Beyotime Biotechnology, Shanghai, China) was used for protein separation and the separated protein was transferred to polyvinylidene fluoride (PVDF) membrane (Millipore, Billerica, MA, USA). Membranes were blocked and probed with primary antibody (anti-KIAA1429, PA5-95717, Thermo Fisher, 1:1000) and then secondary antibody horseradish peroxidase (HRP)-conjugated anti-β-actin (1:2000, Cell Signaling Technology). Blots were visualized with ECL and quantified using Image Studio software.

### Migrative ability assay

Wound healing was performed for the migrative ability assay. In brief, cells were seeded in a six-well plate and grew near 90% confluence. Monolayer cells were manually wounded by a 200-ul pipette tip. After being washed by PBS twice, the monolayer was incubated at 37°C fresh medium. At the indicated time, wound closure images were calculated by the following formula: migration rate = migration distance/original distance.

### Gefitinib-sensitive assay

The drug-sensitivity test of gefitinib was detected using CCK-8 to calculate the half-maximal inhibitory concentration assay (IC_50_). In brief, PC9-GR cells (1.5 × 10^4^ per well) were cultured in 96-well plates with fresh medium. The corresponding concentration of gefitinib (0.1, 0.5, 1, 5, 10, and 20 μM) was administrated to cells. At the indicated time, Cell Counting Kit-8 (Dojindo, Japan) was performed to measure the drug sensitivity using a microplate reader (Thermo Fisher, USA) at 450 nm.

### m^6^A quantification

Total RNA was isolated from NSCLC cells using TRIzol (Invitrogen, CA) according to the manufacturer’s instruction. Besides, the quality was analyzed by NanoDrop 3000. The m^6^A quantification in the total RNA was determined using m^6^A RNA methylation quantification kit (ab185912, Abcam). The m^6^A level was colorimetrically quantified according to the absorbance of each well at 450 nm.

### RNA immunoprecipitation (RIP) assay

NSCLC cells were lysed with lysis buffer (100 mL) containing protease and ribonuclease inhibitors on ice. Centrifugation was carried out for 5 min at 4 °C. The cell lysate was incubated with protein A/G beads coated with anti-KIAA1429-specific and normal IgG antibody with rotation at 4 °C overnight. The experiment was processed using Magna RIP RNA-Binding Protein Immunoprecipitation kit (Millipore, Billerica, MA, USA) instruction. The interaction between KIAA1429 and HOXA1 was verified by qRT-PCR.

### m^6^A−RNA immunoprecipitation assay (MeRIP-qPCR)

MeRIP-qPCR was performed by the methods described previously. In brief, total RNA was isolated from PC9-GR cells treated with gefitinib and then was chemically fragmented into 100−300-nt fragments. The fragments were incubated with m^6^A antibody (ab208577, Abcam, 1:1000) or anti-IgG-conjugated with protein A/G magnetic beads in IP buffer at 4 °C overnight for immunoprecipitation according to the manufacture’s instruction. Total RNA was eluted with elution buffer and purified. Eluated or input total RNA (10 ng) was revers-transcribed using SuperScript First-Stand Synthesis system (Invitrogen, US). The enrichment of m^6^A-precipitated mRNA was calculated by quantitative RT-PCR.

### RNA stability

The HOXA1 mRNA transcription expression was prevented by the addition of Actinomycin D (2 mg/ml) or negative control (DMSO, Sigma-Aldrich, St. Louis, MO, USA). After treatment, RNA expression was determined by qRT-PCR.

### In vivo tumorigenesis

Male BALB/c nude mice (5-week-old) were purchased from Vitalstar Biotechnology Co., Ltd (Beijing, China) to investigate the effects of KIAA1429 on NSCLC tumor growth. This assay was approved by the Ethics Committee of Cancer Hospital of China Medical University. PC9-GR cells stably infected with KIAA1429-targeting shRNA and control were suspended in 100 μL of PBS with Matrigel matrix (BD Biosciences). Then, cells were injected into one of the flanks of BALB/c nude mice. The tumor volumes were measured every 3 days by Vernier caliper and calculated using the formula: (length × width^2^)/2. After 4 weeks, the mice were sacrificed and the weight was weighed.

### Statistical analysis

The patients’ survival curves were analyzed using Kaplan−Meier method with log-rank test. The interaction within clinicopathological features and KIAA1429 expression was analyzed by chi-squared test or Fisher’s test. Intergroup difference was analyzed using Student’s t-test and one-way ANOVA. Statistical analysis was carried out using SPSS software (SPSS, Chicago, IL, USA). *P* < 0.05 was considered statistically significant.

## Supplementary information

Table S1
