# Supplementary material for: N6-methyladenosine (m6A) methyltransferase KIAA1429 accelerates the gefitinib resistance of non-small-cell lung cancer
Source: Cell Death Discov. 2021 May 17;7:108. doi: 10.1038/s41420-021-00488-y (PMC8128911; doi:10.1038/s41420-021-00488-y)
Supplement: Supplementary file 1 — Table S1 [file 41420_2021_488_MOESM1_ESM.docx]

**Supplement Table S1**. Sequences of shRNA and qRT-PCR primers.

|  | 5’-3’ |
| --- | --- |
| KIAA1429 | forward, 5’-AAGTGCCCCTGTTTTCGATAG-3'  reverse, 5'-ACCAGACCATCAGTATTCACCT-3’ |
| HOXA1 | forward, 5’-TCCTGGAATACCCCATACTTAGC-3'  reverse, 5'-GCACGACTGGAAAGTTGTAATCC-3' |
| sh-KIAA1429-1 | 5’-ATTCCGAGCTAATTCAAGATTT-3’ |
| sh-KIAA1429-2 | 5’-TTAAGCGCTACCGGGATTT-3’ |
| sh-KIAA1429-3 | 5’-ACCTGGTTCCAAGCACGCTTTTT-3’ |
| beta-actin | forward, 5’-CTCCATCCTGGCCTCGCTGT-3’  reverse, 5’-GCTGTCACCTTCACCGTTCC-3’ |
